# Seroprevalence of small ruminant brucellosis and owners knowledge, attitude and practices in Chiro and Burka Dhintu Districts, West Hararghe, Ethiopia

**DOI:** 10.1016/j.heliyon.2024.e37708

**Published:** 2024-09-11

**Authors:** Ambachew Motbaynor Wubaye, Shimelis Mitiku, Dagne Tsegaye Lataa, Yihenew Getahun Ambaw, Melkamu Temesgen Mekonen, Simegnew Adugna Kallu

**Affiliations:** a College of Veterinary Medicine, Haramaya University, Dire Dawa, Ethiopia; b Hirna Regional Veterinary Laboratory, Hirna, Ethiopia

**Keywords:** Burka Dhintu, Chiro, I-ELISA, MRBPT, Seroprevalence, Small ruminants, Brucellosis

## Abstract

Brucellosis is one of zoonotic bacterial diseases with significant veterinary and public health consequences in sub-Saharan African countries, including Ethiopia. A cross-sectional study design was conducted with the objective of estimating the seroprevalence of small ruminant brucellosis and assessing owners' knowledge, attitude and practices (KAP) on brucellosis in Chiro and Burka Dhintu Districts in Eastern Ethiopia. A total sample of 444 animals were taken using a cluster based multistage sampling technique. Screening for *Brucella* antibodies and confirmation of positive test results were conducted using Modified Rose Bengal Plate Test (MRBPT) and indirect enzyme-linked immunosorbent assay (I-ELISA) respectively. For the questionnaire survey, 444 randomly selected sheep and goat owners were interviewed using a pretested structured questionnaire. The overall seroprevalence of small ruminant brucellosis was found to be 6.5 % (95 % CI: 4.6–9.3). The multivariable logistic regression analysis identified sex and age as potential risk factors (P < 0.05). More specifically, females were 3.4 times (AOR = 3.4, 95 % CI: 1.2–9.2) more likely to become seropositive than their counterparts, and the odds of seropositivity in adult sheep and goats was 5.6 times (AOR = 5.6, 95 % CI: 1.3–24.7) higher than that of young animals. The knowledge, attitude and practices of animal owners with regard to brucellosis were low, and the level of education was independently associated with the owners’ knowledge and attitude. Moderate seroprevalence, combined with inadequate knowledge, attitudes, and practices of animal owners, makes small ruminant brucellosis a threat to animals and the entire community. Hence, strengthening veterinary services and raising community awareness about the disease is essential to reduce the impact on small ruminant productivity and minimize the risks to public health.

## 1 Introduction

Ethiopia is endowed with the largest small ruminant population in the world, believed to own 42.9 million and 52.5 million sheep and goats respectively, which both account for around 4 % of the world's and 10 % of Africa's small ruminant population [1]. These animals and their products especially meat constitute an important export commodity that significantly contributes to Ethiopia's economy [2]. Though Ethiopia has enormous number of small ruminant population as compared to other countries in Africa, the country does not fully benefited from the sector due to disease and other related factors [3] and brucellosis is one of the infectious diseases that hamper sheep and goats Production in the country [2].

Brucellosis is one of the major zoonotic and a wide spread livestock disease in the world [4]. The disease has a major socio-economic impact in the livelihoods of communities who depend on animal production. The losses due to the disease are associated with abortion, neonatal death, reduced fertility, decreased milk production, costs of preventive measures, and trade restrictions imposed on animals and animal products [5].

Small ruminant brucellosis is caused primarily by *Brucella melitensis* and *B. ovis* and occasionally by *B. abortus* [6]. Sheep and goats acquire the disease through ingestion of feed or water contaminated with infective discharges, or sexual contacts. The zoonotic implication of brucellosis is that it causes chronic debilitating disease in humans with an estimated half a million human cases reported worldwide annually [7]. The main routes of human infections occur mostly through the consumption of contaminated raw animal products and direct contact with contaminated secretions and tissues of infected animals particularly aborted fetus and vaginal discharges [8].

The seroprevalence of small ruminant brucellosis is often impacted by several factors including those pertaining to the animal, agent and environmental [9]. The prevalence of the disease is remarkably high in low-income countries with significant animal and public health concerns, which is mainly attributed to the absence of adequate public health awareness and measures, poor veterinary services, lack of appropriate diagnostic facilities and control strategies [10].

Previous studies in Ethiopia reported the prevalence of brucellosis in small ruminants that include 1.23 % in Somali region [11], 1.76 % in central Ethiopia [12], 3.5 % in Tigray region [13], and 4.8 % in Afar region [2]. These findings indicated variations in the epidemiological patterns of small ruminant brucellosis among different agroecological area. Regardless of a number of studies conducted in the country to establish the seroprevalence of brucellosis in small ruminants, there is lack of a seroprevalence study in small ruminant population in Chiro and Burka Dhintu Districts and scarce information on owners’ knowledge, attitude, and practices (KAP) regarding brucellosis. This highlights the need to investigate the seroprevalence of the disease and assess KAP of sheep and goat owners towards the small ruminant brucellosis in these districts.

## 2 Materials and methods

### 2.1 Study area description

**Fig. 1:**
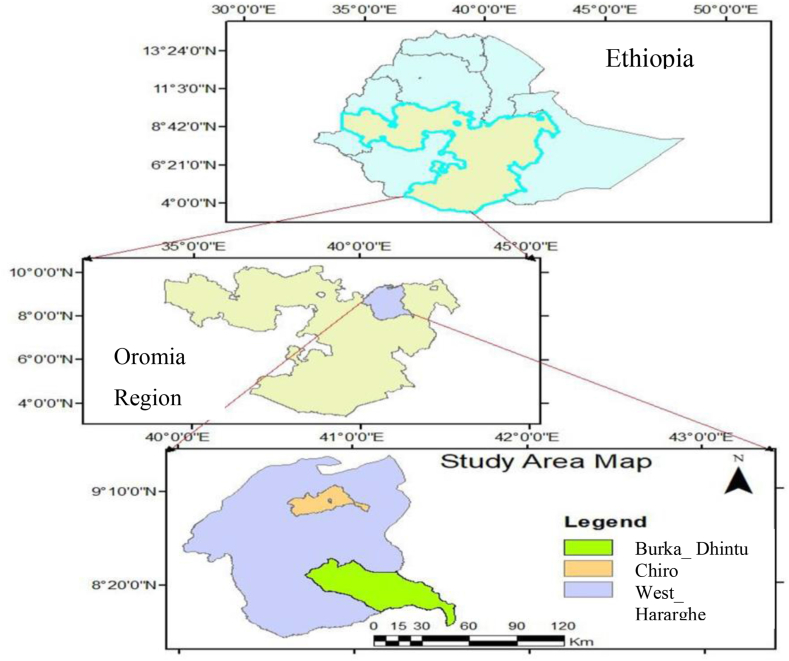
Map showing the study areas (Chiro and Burka Dhintu Districts).

The research was carried out in Burka Dhintu and Chiro Districts of west Hararghe, Eastern Ethiopia (Fig. 1). Burka Dhintu District is found nearly 476 km from Addis Ababa, Ethiopia in the southeastern direction. The district is located within the latitude and longitude of 8°19′N and 40°34′E and it is one among the four pastoral districts in the West Hararghe Zone. The district's agricultural ecology is mainly lowlands having elevations between 700 and 1600 m above sea level. It gets an average of 550 mm–700 mm rainfall per year, and the temperature ranges from 22 °C to 35 °C. Pastoral production systems are mainly practiced in the area and livestock, especially small ruminants, production is a major component of the livelihoods of the community.

Chiro District is found the West Hararghe Zone, nearly 326 km from Addis Ababa, Ethiopia in the eastern direction. The district is located within the latitude and longitude of 8°54′N and 40°14′E. The district is characterized by lowland (700–1600 m) and midland (1600–2200 m) agro-ecology. Chiro District gets an average of 600–1300 mm rainfall per year, and temperature between 17.5 °C and 27.5 °C. Agro-pastoral production systems are mainly practiced in the area. Animals in the extensive management system are grazing freely during day time and kept in inappropriately constructed fenced barns and houses at night and they share grazing and watering points. Few farmers practice semi-intensive management system, in which animals are allowed to graze for some period and then kept in-doors with limited nutritional supplements. Local breeds of sheep and goat reared in Chiro and Burka Dhintu Districts of west Hararghe zone were the study population and all sheep and goats older than four months were eligible to be chosen for this research.

### 2.2 Study design

A cross sectional study design was employed to estimate the seroprevalence, determine the related risk factors of small ruminant brucellosis and to assess knowledge, attitude and practices of owners about brucellosis in Chiro and Burka Dhintu Districts of west Hararghe zone, Ethiopia, from July 2022 to January 2023. To account the production system and agro-ecology for the seroprevalence of brucellosis, two districts which have different production systems (pastoral and agro-pastoral) and agro-ecologies (lowland and midland) were purposively selected. Burka Dhintu has 24 PAs, while Chiro has 39 PAs and each PA has four villages. Therefore, seven PAs from Burka Dhintu and ten PAs from Chiro District were randomly selected. Then, two villages per PAs were selected randomly; hence 34 total villages were selected for the research. The list of PAs and villages were obtained from the administrative office of each district.

### 2.3 Sample size and sampling technique

A single population proportion formula given by Thrusfield [14] as $n=1.96^{2}p_{\exp}\left(1-p_{\exp}\right)/d^{2}$, where, n = the total sample size, p_exp_ = expected prevalence, and d = desired absolute precision, was employed to get the minimum sample size required to estimate sheep and goats brucellosis seroprevalence status in the selected area. Thus, using a 5 % absolute precision, 95 % confidence level, and considering a previous 13.7 % seroprevalence [15], yielded 182 study animals. However, the study employed a multistage sampling strategy, in which the peasant associations (PAs) within the district, villages within the peasant association, and individual animals within the village were the primary, secondary, and tertiary sampling units, respectively. The required sample size was adjusted by multiplying it with the design effect (Deff), to take into account the intra-class correlation coefficient using the formula described by Alimohamadi and Sepandi [16] as: Deff = [1 + ρ(n-1)], where ρ(rho) is the intra-class correlation coefficient, estimated to be 0.029 from a national wide survey of contagious caprine pleuropneumonia in Ethiopia [17], and n is the average small ruminant herd size, estimated to be 43 by the district agricultural office. The calculated Deff becomes 2.218, and multiplying Deff by the sample size obtained from the single population formula above (n = 182) yielded a sample size of 404. Finally, an overall sample size of 444 was obtained, accounting the 10 % contingency for animal owners who may refuse to take part in the research.

### 2.4 Study animals description

Out of 444 sampled animals, 244 were from Burka Dhintu and 200 from Chiro Districts, and among these, 181 and 263 were male and female, respectively. Based on the species of animals, the study animals comprised of 153 sheep and 291 goats. Of all study animals, 279 were managed under an extensive management system, and 165 were managed under a semi-intensive management system. According to the age category, 185 were grouped as adult (above two years of old) and 159 were young (less than or equal to two years of old).

### 2.5 Questionnaire survey and data measurement techniques

To collect relevant data about the knowledge, attitude, and practices towards brucellosis and the hypothesized risk factors for small ruminant brucellosis, a pretested structured questionnaire was administered to 444 randomly selected sheep and goats' owners after translation into a local language (Afaan Oromoo) in face-to-face interviews. The lists of owners were obtained from each PA administrative office. The 444 farmers who participated in the questionnaire survey were the owners of the 444 small ruminants selected for the seroprevalence test. The internal consistency and reliability of the questionnaire were checked in a pilot study with 30 participants who were excluded from the final analysis. The outcome of the pilot study gives a score of Cronbach's alpha 0.737, 0.804, and 0.864 for knowledge, attitude, and practices, respectively, indicating the internal consistency.

There were ten inquiries about the respondents' level of knowledge, seven inquiries about their attitudes, and five inquiries concerning their practices. For each category, if the respondent answered the question correctly, it was given a score of 1; otherwise, it was given a score of 0. After summing all correctly answered scores and using the mean as a cut-off point, the respondents further regrouped into two for each category. Those scoring above or equal to the mean were grouped as having good knowledge, attitudes, and practices, while those who scored below the mean were grouped as having poor knowledge, attitudes, and practices [18].

In the context of this study, the education level of the study subjects was categorized into three as: those attended a formal education and can read and write easily were categorized as educated, while those who did not attend a formal education but can read and write through informal education were categorized as “read and write”. Those who cannot read and write were grouped as “non-educated” categories.

### 2.6 Blood sample collection and serological tests

Approximately 7 mL of blood was drawn from each study animal jugular vein by using plain vacutainer tubes, needle holders, and needles. The blood samples were centrifuged for 3 min at 10,000 rpm after labeling, and sera were extracted by siphoning into sterile cryovials and transported to Hirna regional veterinary laboratory using an ice box, and then the serum samples were refrigerated at −20 °C until serological testing was conducted to identify the existence of anti-Brucella antibodies [13].

Screening was done using Modified Rose Bengal Plate Test (MRBPT) (concentrated suspension of *B. Melitensiss*, Weybridge strain 99; Institut Pourquier, France) and confirmatory test using Indirect Enzyme-linked immune sorbent assay (I-ELISA) (ID Vet, 310, Innovative Diagnosis, France) were conducted at Hirna regional veterinary laboratory using test procedures specified by the World Organization for Animal Health [19] along with the test standards provided by the manufacturer. Briefly, the RBPT was performed by mixing, 75 μl of serum and 25 μl of antigen solution on a glass plate to make a circle that measured about 2 cm in diameter. After that, the mixture was shaken at room temperature (25 °C) for 4 min. Samples exhibiting agglutination were classified as positive, and those without agglutination were classified as negative.

The I-ELISA test was conducted as follows: All wells was filled with190μl of dilution buffer 2 then after 10 μl of negative control was added to A1 and B1 and 10 μl of positive control to C1 and D1. The plate was incubated at 21 °C for 45 min after adding 10 μl of serum sample to all the remaining wells. Then each well was washed with 300 μl of washing solution for three times. The plate was incubated at 21 °C for 30 min after adding 100 μl of conjugate then washed with 300 μl of washing solution for three times. Incubate the plate in the dark room for 15 min after adding 100 μl of the substrate solution. Finally the reaction was stopped by adding 100 μl stop solution. The optical density (OD) was read at 450 nm and documented for all samples. The result was considered negative, if the inhibition percentage was 110 or lower and positive, if the inhibition percentage was 120 or greater. If the inhibition percentage is between 110 and 120, it was regarded as uncertain or doubtful.

### 2.7 Data management and analysis

Laboratory results and questionnaire surveys data were input into and stored in a Microsoft Excel spreadsheet and statistical analysis were computed by using Stata version 16.0. Univariable logistic regression analysis was employed to assess the relationship between small ruminant brucellosis and potential predictor factors. Variables with p < 0.25 were selected as candidates to be included in the multivariable logistic regression analysis in an attempt to control for potential confounding variables, and the adjusted odds ratio was determined. Collinearity between variables was checked by standard error. If the standard errors of the regression coefficients are inflated in the multivariable analysis as compared to univariable analysis, it indicated the possible presence of collinearity, and this further confirmed by the variance inflation factor (VIF). Since VIF is greater than 5, there is no collinearity between variables. The model fitness was determined by the Hosmor-Lemshow test. The Chi-squared (χ^2^) test was used to determine the relationship between potential predictor variables and KAP. Throughout the data presentation p-values less than 0.05 was considered statistically significant.

## 3 Results

### 3.1 Seroprevalence of small ruminant brucellosis

Screening of all samples was done by RBPT and then confirmed by I-ELISA. Out of 444 serum samples, 40 (9.0 %) and 29 (6.5 %) were positive by RBPT and I-ELISA, respectively. The overall seroprevalence of sheep and goat brucellosis in Chiro and Burka Dhintu District was 6.5 %. Based on the univariable logistic regression analysis, factors such as sex, altitude, farming system, management system and age were significantly associated with the seroprevalence of brucellosis sheep and goats (p < 0.05) (Table 1).

**Table 1:** Seroprevalence of sheep and goats brucellosis with the study variables.

Variable	Category	N	MRBPT+	IELISA+	cOR(95 % CI) For I-ELISA	p-value
Districts	Chiro	200	15 (7.5 %)	11 (5.5 %)	1	0.430
	Burka Dhintu	244	25 (10.2 %)	18 (7.4 %)	1.4 (0.6–3.0)	
Species	Sheep	153	12 (7.8 %)	9 (5.9 %)	1	0.690
	Goat	291	28 (9.6 %)	20 (6.9 %)	1.2 (0.5–2.7)	
Sex	Male	181	8 (4.4 %)	5 (2.8 %)	1	0.010^a^
	Female	263	32 (12.2 %)	24 (9.1 %)	3.5 (1.3–9.4)	
Age	Young (≤2 years)	159	5 (3.1 %)	2 (1.3 %)	1	<0.001^a^
	Adult (>2 years)	285	35 (12.3 %)	27 (9.5 %)	8.2 (1.9–35.0)	
Flock size	<20	186	12 (6.5 %)	10 (5.4 %)	1	0.410
	>20	258	28 (10.9 %)	19 (7.4 %)	1.4 (0.6–3.1)	
Farming system	Agro-pastoral	173	6 (3.4 %)	4 (2.3 %)	1	<0.001^a^
	Pastoral	271	34 (12.5 %)	25 (9.2 %)	4.3 (1.5–12.7)	
Altitude	Lowland(<1600m)	260	33 (12.7 %)	24 (9.2 %)	3.6 (1.4–9.7)	0.010^a^
	Midland (1600–2200m)	184	7 (3.8 %)	5 (2.7 %)	1	
Management system	Extensive	279	31 (11.1 %)	26 (9.3 %)	1	<0.001^a^
	Semi-intensive	165	9 (5.5 %)	3 (1.8 %)	0.2 (0.1–0.6)	
Over all prevalence		444	40 (9.0 %)	29 (6.5 %)	4.6%–9.3 %	

N= Number examined; cOR = crude odds ratio.

1 = reference category; MRBPT+ = positive by MRBPT; I-ELISA+ = positive by I-ELISA.

a statistically significant.

### 3.2 Factors independently associated with sheep and goat brucellosis

Sheep and goat brucellosis was found independently associated with sex and age (p < 0.05). More specifically the likelihood of seropositivity for brucellosis in female sheep and goats were 3.4 times (aOR = 3.4, 95 % CI: 1.2–9.2) more than their counter parts. Adult animals were 5.6 times (aOR = 5.6, 95 % CI: 1.3–24.7) more likely to be seropositive for *Brucella* antigen than young animals (Table 2).

**Table 2:** Multivariable logistic regression analysis of potential risk factors of brucellosis.

Variable	Category	N	I-ELISA+ (%)	aOR (95 % CI)	p-value
Sex	Male	181	5 (2.8)	1	0.01^a^
	Female	263	24 (9.1)	3.4 (1.2–9.1)	
Age	Young	159	2 (1.3)	1	0.02∗
	Adult	285	27 (9.5)	5.6 (1.3–24.7)	
Farming system	Agro-pastoral	173	4 (2.3)	1	0.32
	Pastoral	271	25 (9.2)	2.9 (0.4–23.5)	
Altitude	Lowland	260	24 (9.2)	0.75 (0.1–5.4)	0.78
	Midland	184	5 (2.7)	1	
Management	Extensive	279	26 (9.3)	0.3 (0.08–1.0)	0.051
System	Semi-intensive	165	3 (1.8)	1	

N = Number, aOR = adjusted odds ratio.

1 = reference category; I-ELISA+ = positive by I-ELISA.

a = statistically significant.

### 3.3 Knowledge, attitude, and practices of the owners towards small ruminant brucellosis

#### 3.3.1 Knowledge of the respondents towards small ruminant brucellosis

Out of the total respondents, only 24.1 % had heard about brucellosis (Table 3) and the majority of them (50.5 %) were getting the information from veterinarians. From the respondents, only 21.5 % were aware that brucellosis is a zoonotic disease and more than three-quarters (79.4 %) did not know the means of transmission from animals to humans (Table 3).

**Table 3:** Respondents’ knowledge regarding small ruminant brucellosis.

Knowledge related items	Response	Frequency	Proportion
Have you heard of the disease called brucellosis?	Yes	107	24.1
	No	337	75.9
Do you know that brucellosis affects sheep and goats?	Yes	25	23.4
	No	82	76.6
Do you know how brucellosis spreads between animals?	Yes	16	15.0
	No	91	85.0
How spread occurs between animals?	Sexual contact	7	6.5
	Contacts with discharges	3	2.8
	Contaminated feed	5	4.7
	Contaminated water	1	0.9
	Don't know	91	85.1
Have you observed clinical signs like hygroma/swelling of joint/testicles in animals?	Yes	23	21.5
	No	84	78.5
Is there any treatment for brucellosis?	Yes	18	16.8
	No	89	83.2
Do you know that brucellosis is a zoonotic disease?	Yes	23	21.5
	No	84	78.5
Do you know how humans can be infected with brucellosis from sheep or goats?	Contact with diseased animals and their discharges.	4	3.7
	Feeding of raw milk and milk products.	11	10.3
	Feeding of raw or undercooked meat.	7	6.6
	Don't know.	85	79.4
Does brucellosis cause abortion in sheep and goats?	Yes	47	43.9
	No	60	56.1
Do you know the gestation period during which brucellosis causes abortion?	Less than 2 months	16	15.0
	2–3 months	14	13.1
	Greater than 3 months	20	18.7
	Don't know	57	53.3

The mean knowledge score was 2.59 ± 2.99, with a minimum zero score and maximum 10 score. Overall, only 16.8 % have good knowledge about brucellosis (Table 4). From the studied factors, respondent's educational status was found significantly associated with their knowledge (p < 0.01).

**Table 4:** Association between knowledge and respondents’ socio-demography.

Variable	Category	N	Knowledge level (%)		χ^2^	p value
			Good	Poor		
Districts	Burka Dhintu	59	12 (20.1 %)	47 (79.9 %)	1.162	0.281
	Chiro	48	6 (13.5 %)	42 (86.5 %)		
Gender	Male	65	12 (18.1 %)	53 (81.9 %)	0.318	0.573
	Female	42	6 (5.6 %)	36 (84.4 %)		
Age	18–30	30	2 (7.3 %)	28 (93.3 %)	3.132	0.209
	30–45	61	13 (21.0 %)	48 (78.7 %)		
	>45	16	3 (19.4 %)	13 (81.3 %)		
Educational Status	Non educated	68	2 (2.9 %)	66 (97.1 %)	47.865	<0.001^a^
	Read and write	27	6 (22.2 %)	21 (77.8 %)		
	Educated	12	10 (83.3 %)	2 (16.7 %)		
Farming System	Agro-pastoral	45	8 (17.8 %)	37 (82.2 %)	0.05	0.822
	Pastoral	62	10 (16.1 %)	52 (83.9 %)		
Over all		107	18 (16.8 %)	89 (83.2 %)		

N = number of respondents; χ^2^ = Chi-square.

a = statistically significant.

### 3.4 Attitude of respondents on small ruminant brucellosis

From the total interviewed respondents, 13.1 % of them believe that brucellosis is an important public health disease and 16.8 % and 20.6 % of the respondents believes that boiling milk and cooking meat prevents brucellosis, respectively (Table 5).

**Table 5:** Respondents attitude towards small ruminant brucellosis.

Attitude related items	Response	Frequency	Proportion
Do you believe that brucellosis is an important public health concern?	Yes	14	13.1
	No	93	86.9
Do you believe that any family members are at risk of contracting brucellosis? Risk of acquiring brucellosis?	Yes	13	12.1
	No	94	87.9
Which family members are most susceptible to brucellosis?	Don't know	94	87.9
	Children	4	3.7
	Female	8	7.5
	Male	1	0.9
Would you like to receive more information about brucellosis?	Yes	75	70.1
	No	32	29.9
In which method that wants to receive information?	Don't wanted	32	29.9
	Meeting in village	54	50.5
	Educational booklet	2	1.9
	Veterinarian	18	16.8
	Television/Radio	1	0.9
Do you consider brucellosis in sheep/goat a serious disease?	Don't know	82	76.6
	Not serious	5	4.7
	Quite serious	8	7.5
	Very serious	12	11.2
Do you believe boiling milk prevent brucellosis?	Yes	18	16.8
	No	89	83.2
Do you think cooking meat prevent brucellosis?	Yes	22	20.6
	No	85	79.4

The mean attitude score was 1.13 ± 1.12. The total percentage of participants with a desirable attitude towards sheep and goats brucellosis was 20.6 %. Educated respondents showed better desirable attitude (91.7 %) than those who can read and write (37.0 %) non educated (1.5 %) respondents towards brucellosis (p < 0.01) (Table 6).Practice of respondents on small ruminant brucellosis.

**Table 6:** Association between attitude and respondents'socio-demography and small ruminant management.

Variable	Category	N	Attitude level		χ2	P value
			Desirable (%)	Undesirable (%)		
Districts	Chiro	48	13 (27.1)	35 (72.9)	2.267	0.132
	Burka Dhintu	59	9 (15.3)	50 (84.7)		
Gender	Male	65	15 (23.1)	50 (76.9)	0.642	0.423
	Female	42	7 (16.7)	35 (83.3)		
Age	18–30	30	4 (13.3)	26 (86.7)	1.598	0.450
	30–45	61	15 (24.6)	46 (75.4)		
	>45	16	3 (18.7)	13 (81.3)		
Educational Status	Non educated	68	1 (1.5)	67 (98.5)	56.806	<0.001^a^
	Read and Write	27	10 (37.0)	17 (63.0)		
	Educated	12	11 (91.7)	1 (8.3)		
Farming System	Agro-pastoral	45	12 (26.7)	33 (73.3)	1.773	0.183
	Pastoral	62	10 (16.1)	52 (83.9)		
Over all		107	22 (20.6)	85 (79.4)		

N = Total number, χ2 = Chi-square.

a = statistically significant.

Less than half of the respondents (41.0 %) were using milk for human consumption and the majority of them were consuming raw milk (82.4 %). Most of the respondents (92.8 %) assist their sheep/goat during kidding and lambing, but more than three-quarters (74.0 %) of them assist without using personal protective equipment. The majority of the participants (86.9 %) dispose the aborted/dead fetuses dropping to open dumps or giving to dogs (Table 7).

**Table 7:** Respondents’ practices regarding small ruminant brucellosis.

Practice related items	Response	Frequency	Proportion
Do you consume sheep/goat milk	Yes	182	41.0
	No	262	59.0
How do you consume sheep/goat milk for household?	Raw milk	150	82.4
	After boiling	32	17.6
Have you wash your hands before milking?	Yes	18	9.9
	No	164	90.1
Have you wash your hands after milking?	Yes	76	41.8
	No	106	58.2
Do you assist sheep and goats during delivery?	Yes	412	92.8
	No	32	7.2
If you assist sheep and goats during delivery and do you use personal protective equipment?	Yes	107	26.0
	No	305	74.0
How do you dispose aborted and or dead fetuses?	Burning/burying	58	13.1
	Dispose to open dump/giving to dogs	386	86.9

## 4 Discussion

Fundamental element in disease surveillance and monitoring programs to implement control measures comprises understanding the distribution of a disease and public KAPs towards a disease [20].

The present study revealed an overall seroprevalence of 6.5 % using I-ELISA, which is considered as a more sensitive and specific test for the detection of anti-Brucella antibodies. Our observed seroprevalence of 6.5 % is in agreement with the 6.4 % in Korahey zone of Somali regional state, eastern Ethiopia [11], 8.0 % in the Borana Zone, Ethiopia [21], and 5.8 % in Karnataka in India's Southern Province [22]. The comparisons in these findings can be justified by the nearly similar management systems of animals in these study areas, where animals are usually managed extensively in communally shared open fields, grazing areas, watering points and night enclosures. The agro-ecological situations of the study area, particularly, of Burka Dhintu are similar to those of the southern Ethiopia, comprising Borana and Guji. However, the prevalence of small ruminant brucellosis in the current study is higher than previous reports from different parts of Ethiopia, with 0.2 % in West Hararghe Zone [23], 0.7 % around Kombolcha [24], and 1.5 % in South Wollo Zone [23]. The animal management system in these areas with low figures may be due to mixed farming agricultural system with only small number of animals reared separately [24], which could reduce disease transmission. Such animal farming system is different from that of the current study area where communities practice pastoralism and to some extent agro-pastoralist that could probably contributed to the differences in seroprevalence. The seroprevalence of 12.35 % in Ewa and Chifra districts and 13.7 % in Tellalak District recorded respectively by Tegegn et al. [25] and Tadeg et al. [26] were almost two fold higher than the present result. These studies were conducted in the Afar region, which is known for pastoralism [25,26]. These pastoralists move place to place for searching of available water sources and grass for their animals. These movements lead to higher animal densities especially in key areas such as watering points and increasing disease transmission. Drought and dry seasons, which are common climatic factors in many pastoral areas, result in severe animal stress and increase animal susceptibility to diseases [27]. Afar pastoralists also engage in the animal market with West Hararghe zone communities, which could contribute to the relatively moderate seroprevalence of the disease in the current study area. These practices of pastoralism and communal grazing, together with a inadequate veterinary and public health services, can complicate small ruminant brucellosis to remain untreated and endemic [10,27].

The current finding showed adult sheep and goats were 5.6 times more likely to become seropositive for brucellosis than the animals in the young age category. Similar results have been reported previously by Natesan et al. [21], Hussen et al. [11], and Edao et al. [28]. This could be because, even if animals become infected with *Brucella* at a young age, the organism localizes itself in regional lymph nodes without eliciting antibody production until the animal reaches sexual maturity and begin to produce sex hormones and erythritol, which stimulate the growth and multiplication of *Brucella* species [29].

With regard to sex of animals, statistically significant difference (*p* < 0.05) between male and female small ruminant seropositivity was noted. Females were 3.4 times more seropositive than male animals. This finding is in accordance with the finding of Ferede et al. [24] conducted in Bahir Dar and around. The higher seroprevalence of brucellosis in females compared to male animals may be attributed to age of animals, as males are often sold at early age, and young male animals are dominant than adult males. In addition, it may be linked to the hormone erythritol. Erythritol is a sugar alcohol that is found in higher amount in the placenta and fetal fluids of animals with pregnancy, creating an ideal environment for *Brucella* species growth and multiplication. Females also have a higher concentration of erythritol sugar than males, which makes them more susceptible to the disease [29]. Birth of unthrifty neonates, infertility, and abortion, the principal manifestation of brucellosis in females will significantly affect the reproductive potential of females and the expected number of healthy offspring [29].

It was evident from the current questionnaire survey that the community in the study area had inadequate knowledge regarding brucellosis. This finding is consistent with 15 % knowledge in South Eastern Somali Region [30] and higher than null awareness in selected districts of southern region [5] and Jigjiga District, Somali Regional State, Ethiopia [31]. However, it is lower than the 56.8 % reported in Kenya [32] and 70.5 % in Saudi Arabia [33]. The study revealed that only 21.5 % of the survey respondents were aware of the zoonotic implications of brucellosis, which is higher than the 4.4 % in Afar [34] and 2.6 % in Sri Lanka [35]. But it is lower than the 77.6 % in Saudi Arabia [33], 90 % in Kenya [32], and 96.3 % in Egypt [36]. This suggests that brucellosis awareness can differ significantly from region to region and person to person. The differences in brucellosis awareness reported in different studies may be attributed to differences in educational facilities, health extension services, culture, and general public health awareness across the study areas. For example, the higher knowledge and awareness in Saudi Arabia (63.6 %) was attributed to the participants' higher average education level. In Kenya, the study was conducted in Kiambu County, which neighbors Nairobi, the capital city of Kenya, which has good infrastructure with accessible medical and veterinary services [32].

Nearly three quarters (74.0 %) of the study respondents stated that they had provided assistance to their ewes and does during the time of giving birth without using protective equipment. Dosa et al. [5] have reported similar findings in southern region of Ethiopia. Human brucellosis may spread further if animals are assisted without using personal protective equipment and aborted tissue and retained placenta are handled improperly [37]. Therefore, through direct contact with contaminated materials, there is a substantial danger of disease transfer between animals and from animals to humans.

About 86.9 % of the respondents disposed the aborted fetuses either by giving them to dogs or throwing them to open dumps. The result is comparable to a report in Egypt [38], and highlights that, feeding aborted fetuses to dogs may increases the risk of the disease transmission and persistence in the flock since dogs often drag aborted fetus and fetal materials across the ground may and contribute to mechanical transmission of brucellosis [39]. Additionally, the risk of the disease spreading to people and animals is increased when aborted materials are disposed of in open landfills and waterways [40].

Majority of the respondents (82.4 %) consume raw milk, which is similar to the 79 % in Kenya [32], 94.5 % [5] and 97.6 % [34] in the southern and Afar regions of Ethiopia. The habit of raw milk consumption is a risk factor for the transmission of brucellosis from animal to human and is attributed to the belief of animal owners that raw goat milk possesses better taste and various medicinal values including properties to improve sexual desire and performance [32].

The respondents’ knowledge and attitude regarding brucellosis were found associated with their education level (*p* < 0.001), which is similar with the study conducted by Harbi et al. [33]. The results showed that lack of education is an important risk factor associated with having poor knowledge regarding brucellosis. Due to the fact that knowledge influences attitudes, which in turn influence behaviors, educational initiatives and awareness raising campaigns are essential to reduce risky practices for *Brucella* transmission that are observed in the current study area.

### 4.1 Limitations of the study

Although a pilot study was done on 30 participants and attempts were made that the respondents understood questions correctly before they responded, honesty and recall ability of individuals were not independently assessed due to lack of standardized instruments. Additionally, the cause-and-effect relationship might be affected due to the nature of the currently used study design (cross-sectional).

## 5 Conclusions

The current serosurvey investigation revealed that brucellosis is moderately prevalent among small ruminant population. Sex and age were found to be a risk factor for the brucellosis. The KAP of animal owners towards brucellosis in the study area were found low and level of education was found significantly associated with this low level of KAP. Therefore, since people in Ethiopia, particularly in Burka Dhintu and Chiro Districts live close to their animals, which occasionally share housing enclosures, brucellosis poses a serious health risk to the whole community. Hence, it is imperative to raise awareness of brucellosis among animal owners and to motivate behavioral changes regarding the handling of animals during abortion and parturition. Further, large-scale epidemiological research employing one health approach is necessary to identify and characterize circulating *Brucella* species among humans and livestock so as to identify the transmission dynamics of the organism.

## Ethical statement

This research was approved by Haramaya University, College of Veterinary Medicine Ethical Clearance Committee (Ref. No: CVM/796/2022). Each participant who decided to participate in this study provided written informed consent, and the information collected was kept confidential and utilized solely for this research.

## Data availability statement

All data will be made available upon reasonable request to and with the approved consent of the corresponding author, and will be shared in accordance with the standards of ethical policies regulating data sharing in human subjects.

## CRediT authorship contribution statement

**Ambachew Motbaynor Wubaye:** Writing – original draft, Visualization, Methodology, Formal analysis, Conceptualization. **Shimelis Mitiku:** Writing – review & editing, Writing – original draft, Methodology, Conceptualization. **Dagne Tsegaye Lataa:** Writing – review & editing, Writing – original draft, Supervision, Conceptualization. **Yihenew Getahun Ambaw:** Writing – review & editing, Formal analysis, Data curation. **Melkamu Temesgen Mekonen:** Writing – review & editing, Supervision, Conceptualization. **Simegnew Adugna Kallu:** Writing – review & editing, Methodology, Formal analysis, Conceptualization.

## Declaration of competing interest

The authors declare no competing interests.
