# Supplementary material for: Seroprevalence of small ruminant brucellosis and owners knowledge, attitude and practices in Chiro and Burka Dhintu Districts, West Hararghe, Ethiopia
Source: Heliyon. 2024 Sep 11;10(18):e37708. doi: 10.1016/j.heliyon.2024.e37708 (PMC11422036; doi:10.1016/j.heliyon.2024.e37708)
Supplement: Multimedia component 1 [file mmc1.docx]

**Section I: Demographic data**

1. Age (Years): ____________
2. Gender: ___________
3. District _________
4. Level of education:
5. Non educated B. Read and write D. Educated
6. Farming system ___________________

**Section II: Questions to assess participants’ Knowledge about brucellosis**

1. Have you heard of the disease called brucellosis?

A. Yes B. No

1. Where did you get information about brucellosis?
2. Veterinarians B. Health workers C. Media
3. Do you know that brucellosis affects sheep and goats?
4. Yes B. No
5. Have you observed clinical signs like hygroma/swelling of joint/testicles in animals?
6. Yes B. No
7. Do you know how brucellosis spreads between animals?
8. Yes B. No
9. How spread occurs between animals?
10. Sexual contact B. Contact with discharges C. Contaminated feed

D. Contaminated water E. Do not know

1. Is there any treatment for brucellosis?
2. Yes B. No
3. Do you know that brucellosis is a zoonotic disease?
4. Yes B. No
5. Do you know how humans can be infected with brucellosis from sheep or goats?
6. Close contact with infected animals and discharges
7. Consumption of raw milk and milk products
8. Consumption of raw or undercooked meat
9. Don’t know
10. Does brucellosis cause abortion in sheep and goats?
11. Yes B. No
12. Do you know the gestation period during which brucellosis causes abortion?
13. < 2 months
14. months
15. > 3 months
16. Don’t know

**Section III: Questions to assess participants’ Attitude about brucellosis**

1. Do you believe that brucellosis is an important public health concern?
2. Yes B. No
3. Do you believe that any family members are at risk of contracting brucellosis? Risk of acquiring brucellosis?
4. Yes B. No
5. Which family members are most susceptible to brucellosis?
6. Don’t know B. Children C. Female D. Male
7. Would you like to receive more information about brucellosis?
8. Yes B. No
9. In which method that wants to receive information?
10. Don’t wanted B. Meeting in village C. Educational booklet

D. Veterinarian E. Television/Radio

1. Do you consider brucellosis in sheep/goat a serious disease?
2. Don’t know B. Not serious C. Quite serious D. Very serious
3. Do you believe boiling milk prevent brucellosis?
4. Yes B. No
5. Do you think cooking meat prevent brucellosis?
6. Yes B. No

**Section IV: Questions to assess participants’ Practices towards brucellosis**

1. Do you consume sheep/goat milk?
2. Yes B. No
3. How do you consume sheep/goat milk for household?
4. Raw milk B. After boiling
5. Have you wash your hands before milking?
6. Yes B. No
7. Have you wash your hands after milking?
8. Yes B. No
9. Do you assist sheep and goats during delivery?
10. Yes B. No
11. If you assist sheep and goats during delivery, do you use personal protective equipment?
12. Yes B. No
13. How do you dispose aborted and or dead fetuses?
14. Burning/burying B. Dispose to open dump/giving to dogs
